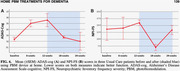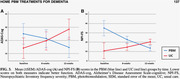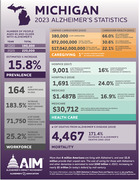# Providing In‐Home Helmet‐Based 40 Hz Light Therapy for Alzheimer’s Patients in Memory Care Facilities in Detroit, MI: A Proposal for a Feasibility Study to Expand Early Efficacy Data

**DOI:** 10.1002/alz.094270

**Published:** 2025-01-09

**Authors:** Jacob A Bart, Zechariah Attar

**Affiliations:** ^1^ Henry Ford Heath, Detroit, MI USA; ^2^ Henry Ford Health, Detroit, MI USA

## Abstract

**Introduction:**

Alzheimer’s disease is the most common and costly neurodegenerative disease worldwide. Early studies of helmet‐based 40 Hz light therapy have demonstrated safety and early efficacy in targeting the pathophysiologic characteristics of Alzheimer’s disease. This intervention has shown slowing and even reversal of disease progression. An extensive literature review has demonstrated this therapy to be cost‐effective and user‐friendly with minimal side effects. Implementation of this therapy has been limited by barriers to access, illustrated by small sample sizes and poor compliance in numerous studies. Further research with larger sample sizes is essential to accelerate the rate of translation from bench to bedside by addressing implementation barriers to access, thereby optimizing patient adherence.

**Hypothesis:**

We hypothesize that offering helmet‐based 40 Hz light therapy in a home setting will result in increased enrollment of eligible patients and compliance by providing convenience to patients and their caregivers. This approach will develop implementation guidelines while expanding on existing efficacy data.

**Objective:**

To explore our hypothesis, we plan to conduct a feasibility study by recruiting through, and providing care at, memory care facilities in Detroit, Michigan. Changing the location of care may be an easily adaptable implementation strategy that can be applied nationally. We've identified 50 memory care facilities that house Alzheimer’s patients, with capacity for over 400 residents in Detroit alone.

**Methods:**

We plan to assemble a mobile team of research‐clinicians to administer helmet‐based 40 Hz light therapy. Scheduled weekly appointments can be facilitated at the convenience of the patients and their care providers across all facilities that allow this therapy for their residents. These participants will be recruited via rolling admission and outcomes will be evaluated using standardized neurocognitive assessments. In summary, helmet‐based 40 Hz therapy has already been shown to be safe and effective. Studying the feasibility of implementing it in the patient’s home is an important next step in making this care accessible to those that need it most.